# Synchronous hepatocellular carcinoma and nasopharyngeal carcinoma in a patient with chronic hepatitis B: A case report and literature review

**DOI:** 10.1097/MD.0000000000051434

**Published:** 2026-11-06

**Authors:** Shijie Zhang

**Affiliations:** a Department of Interventional Vascular Surgery, Guangxi Academy of Medical Sciences, People’s Hospital of Guangxi Zhuang Autonomous Region, Nanning, Guangxi, China.

**Keywords:** case report, hepatocellular carcinoma, immunotherapy, nasopharyngeal carcinoma, synchronous double primary cancer

## Abstract

**Rationale:**

Synchronous double primary cancers involving hepatocellular carcinoma (HCC) and nasopharyngeal carcinoma (NPC) are exceptionally rare. Chronic hepatitis B virus (HBV) infection is an established cause of HCC, whereas Epstein–Barr virus (EBV) infection is the major etiological factor for NPC. We report a patient with synchronous HCC and NPC and review the relevant literature.

**Patient concerns:**

A 63-year-old Chinese man with chronic HBV presented with a liver mass detected on routine screening. He reported nasal obstruction.

**Diagnoses:**

Contrast-enhanced imaging showed a 46 × 59 mm hepatic lesion with portal vein tumor thrombus and a 41 × 29 mm nasopharyngeal mass with bilateral cervical lymphadenopathy. Biopsies confirmed moderately differentiated HCC and EBV-associated non-keratinizing NPC. Final diagnoses were HCC Barcelona Clinic Liver Cancer Stage C (CNLC IIIa, Child-Pugh A) and NPC T3N2M0 Stage III (AJCC 8th edition).

**Interventions:**

He received hepatic arterial infusion chemotherapy (HAIC, mFOLFOX) combined with tislelizumab and donafenib; radiotherapy for NPC was deferred after shared decision-making. Entecavir was continued.

**Outcomes:**

At 19-month follow-up, both tumors remained controlled; the NPC met radiographic complete response criteria.

**Lessons:**

Synchronous HCC and EBV-associated NPC were controlled during treatment with a tislelizumab-containing multimodality regimen, and the NPC achieved a radiographic complete response. The contribution of each treatment component cannot be determined from a single case, and definitive radiotherapy remains the standard of care for locoregionally advanced NPC. Multidisciplinary management and close monitoring are essential.

## 1. Introduction

Multiple primary malignant neoplasms refer to 2 or more histologically distinct malignancies in the same individual, classified as synchronous (diagnosed within 6 months) or metachronous (>6 months apart).^[1,2]^ The incidence of multiple primary malignant neoplasms has risen with improved cancer survival, aging populations, and greater diagnostic sensitivity. However, synchronous hepatocellular carcinoma (HCC) and nasopharyngeal carcinoma (NPC) have been documented in only 2 cases in the English-language literature.^[3,4]^

HCC is the sixth most common cancer worldwide and the third leading cause of cancer-related death.^[5]^ In East Asia, chronic hepatitis B virus (HBV) infection accounts for approximately 80% of cases.^[6]^ NPC shows markedly elevated incidence in Southern China. Epstein–Barr virus (EBV) infection, genetic susceptibility, and environmental factors are established risk factors.^[7,8]^

Epidemiological studies have reported associations between chronic HBV infection and certain extrahepatic cancers, but the evidence for a causal role of HBV in NPC is limited and indirect.^[9,10]^ We report a case of synchronous HCC and EBV-associated NPC in an HBV-infected patient and review the relevant literature, focusing on the diagnostic and multidisciplinary treatment challenges.

## 2. Case presentation

### 2.1. Patient information

A 63-year-old Chinese man was admitted on December 19, 2023, 4 days after a liver mass was detected on routine screening ultrasonography. He reported nasal obstruction but denied epistaxis, hearing impairment, cervical swelling, weight loss, fever, and abdominal pain. He had chronic hepatitis B without regular antiviral therapy. He denied hypertension, diabetes, smoking, or alcohol use, and had no family history of malignancy. He had no occupational exposure to formaldehyde, wood dust, welding/chemical fumes, or combustion exhaust.

### 2.2. Physical examination

The patient was alert and oriented. No palpable superficial lymphadenopathy was detected. Cardiopulmonary and abdominal examinations were unremarkable. The liver and spleen were not palpable, and no lower extremity edema was present.

### 2.3. Laboratory investigations

Complete blood count showed white blood cells 6.52 × 10^9^/L, hemoglobin 124.0 grams/L, and platelets 121 × 10^9^/L. Liver function tests showed aspartate aminotransferase 35 U/L, alanine aminotransferase 24 U/L, total bilirubin 21.7 μmol/L, direct bilirubin 5.5 μmol/L, and serum albumin 28.1 grams/L. Prothrombin time was mildly prolonged (Child-Pugh score 1); baseline INR was not separately recorded. Virological studies showed HBsAg positive, HBeAg positive, anti-HBc positive, anti-HBs negative, and baseline HBV-DNA 4.3 × 10^5^ IU/mL. Serum EBV antibodies (VCA-IgA and EA-IgA) were negative. Alpha-fetoprotein was >1210.00 μg/L (reference < 7.0 μg/L), and protein induced by vitamin K absence-II was 147.00 mAU/mL (reference < 40 mAU/mL). The baseline Child-Pugh score was 6 (class A): hepatic encephalopathy 1, ascites 1, total bilirubin 1, serum albumin 2, and prothrombin time prolongation 1. Imaging before treatment showed features of cirrhosis and mild ascites.

### 2.4. Imaging and histopathology

Contrast-enhanced computed tomography revealed a 46 × 59 mm hepatic mass in the right lobe with arterial hyperenhancement and portal venous washout. Portal vein tumor thrombus involving the right portal vein was identified (Fig. 1A). The liver had a nodular contour consistent with cirrhosis, with mild perihepatic ascites. Nasopharyngeal magnetic resonance imaging (MRI) showed a 41 × 29 mm soft tissue mass in the posterior-superior wall with invasion of the bilateral levator veli palatini and tensor veli palatini muscles, and the clivus. Bilateral cervical lymphadenopathy at levels I, II, and III was consistent with nodal metastases; the largest node measured 22 × 18 mm, did not extend below the caudal border of the cricoid cartilage, and showed no radiological extranodal extension (Fig. 1B).

**Figure 1.**
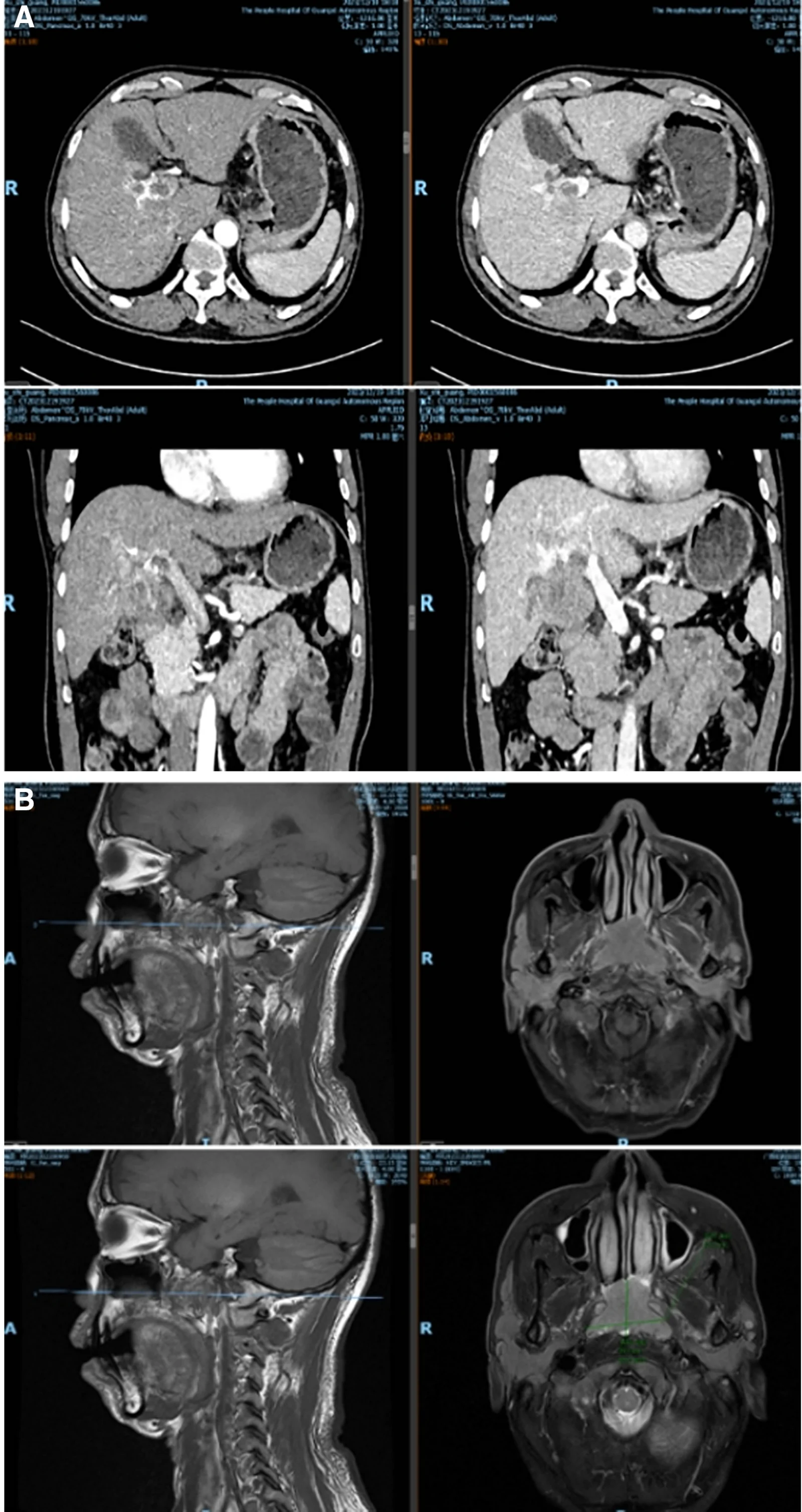
Initial imaging at diagnosis. (A) Contrast-enhanced liver CT demonstrating a 46 × 59 mm hypervascular mass in the right hepatic lobe with portal vein tumor thrombus (arrow). (B) Nasopharyngeal MRI showing a 41 × 29 mm soft tissue mass (asterisk) in the posterior-superior nasopharyngeal wall with bilateral cervical lymphadenopathy (arrows). CT = computed tomography, MRI = magnetic resonance imaging.

Liver biopsy revealed moderately differentiated HCC with trabecular architecture (Fig. 2A). Immunohistochemistry (IHC): Glypican-3+, HepPar-1 (partial)+, CD34 (sinusoidal vascularization), Ki-67 ~40%. P40, CK7, and synaptophysin were negative. Epstein–Barr virus-encoded small RNA (EBER) in situ hybridization was negative. Reticulin staining confirmed disrupted hepatic plate architecture.

**Figure 2.**
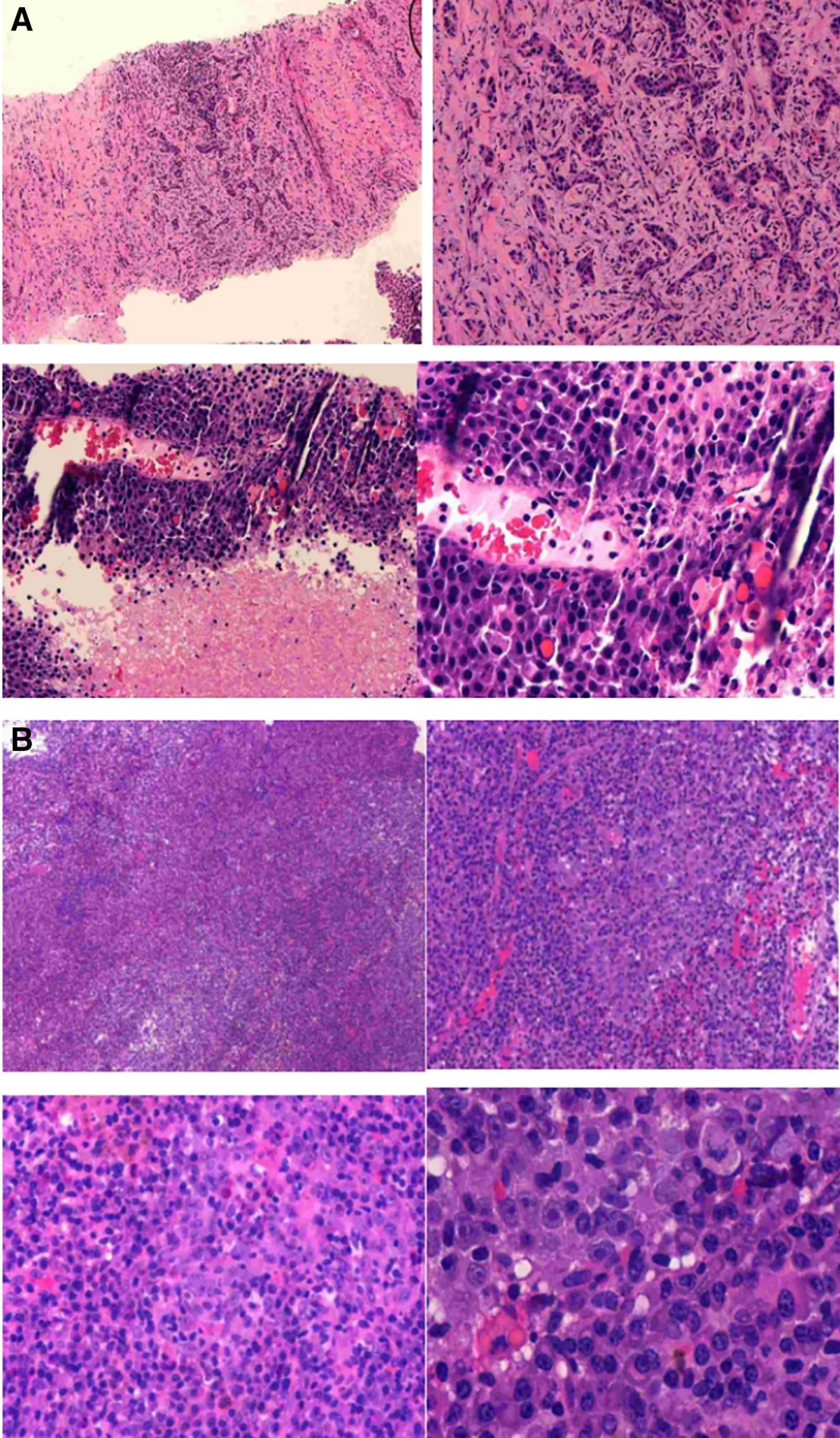
Histopathological findings. (A) Liver biopsy: moderately differentiated HCC with trabecular architecture (H&E, ×200). Tumor cells are positive for Glypican-3 and HepPar-1, with CD34 highlighting sinusoidal vascularization. In situ hybridization for EBER is negative. (B) Nasopharyngeal biopsy: EBV-associated non-keratinizing SCC, undifferentiated subtype (H&E, ×200). Tumor cells are positive for P40, P63, and CK5/6. EBER in situ hybridization is diffusely positive in tumor nuclei. HCC = hepatocellular carcinoma, EBER = Epstein–Barr virus-encoded small RNA, EBV = Epstein–Barr virus.

Nasopharyngeal biopsy showed EBV-associated non-keratinizing squamous cell carcinoma, undifferentiated subtype (Fig. 2B). IHC: P40+, P63+, EGFR+, CK5/6+, Ki-67 ~60%. P16, CD56, CD20, and CD3 were negative. EBER in situ hybridization was diffusely positive, confirming EBV-associated NPC. Programmed death-ligand 1 (PD-L1) immunohistochemistry was not performed.

### 2.5. Final diagnosis

Primary HCC: Barcelona Clinic Liver Cancer Stage C, CNLC Stage IIIa, Child-Pugh Class A; NPC with bilateral cervical lymph node metastases: T3N2M0, Stage III (AJCC 8th Edition); HBV-related cirrhosis (compensated; Child-Pugh class A); Chronic HBV carrier.

### 2.6. Treatment plan

After multidisciplinary team discussion, a comprehensive integrated treatment plan was formulated:

- HCC: Hepatic arterial infusion chemotherapy (HAIC) (mFOLFOX) combined with tislelizumab 200 mg IV q3w and donafenib 200 mg bid po.
- NPC: Definitive radiotherapy-based treatment was planned after initial systemic therapy response assessment. Because the NPC met radiographic complete response criteria, the radiation oncologist recommended continuing immunotherapy with active surveillance rather than immediate radiotherapy; the patient and family agreed after shared decision-making and counseling. Salvage radiotherapy was reserved for local recurrence. Additional systemic intravenous chemotherapy for NPC was not administered because of concerns about tolerability after HAIC.
- Antiviral: Entecavir 0.5 mg daily.

### 2.7. Clinical course

After multidisciplinary team discussion (hepatology, radiation oncology, medical oncology, interventional radiology), curative resection was deemed infeasible.

Cycle 1 HAIC was administered on December 21, 2023 (Fig. 3A), followed by tislelizumab and donafenib. Pre-radiotherapy evaluation on January 3, 2024, showed a radiographic complete response of the NPC primary and cervical nodes. The radiation oncologist recommended continuing immunotherapy with active surveillance and deferring radiotherapy; the patient and family agreed after counseling about the risks of deferral and the availability of salvage radiotherapy.

**Figure 3.**
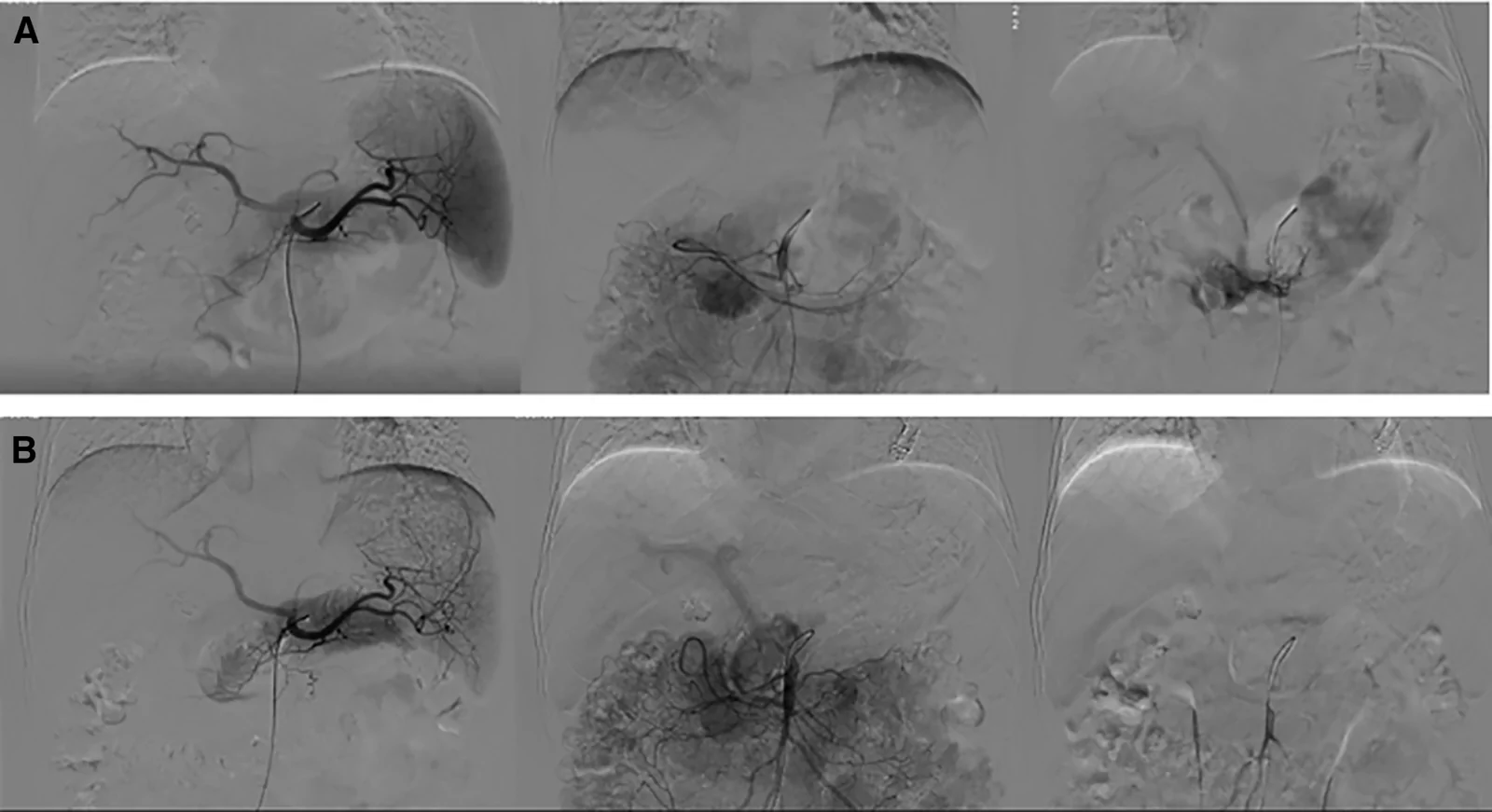
HAIC digital subtraction angiography. (A) First cycle showing tumor supply from a superior mesenteric artery branch with a filling defect in the right portal vein (arrow). (B) Second cycle demonstrating reduced tumor vascularity and improved portal vein filling. HAIC = hepatic arterial infusion chemotherapy.

Cycle 2 HAIC was performed on January 15, 2024 (Fig. 3B). Digital subtraction angiography showed reduced tumor vascularity and improved portal vein thrombus.

After cycle 2, the patient developed Grade 2 ascites, hypoalbuminemia, and Grade 2 thrombocytopenia. Imaging on February 19, 2024, confirmed a sustained radiographic complete response of the NPC. After shared decision-making, the family elected to defer radiotherapy. Ultrasound-guided paracentesis on February 29, 2024, yielded ascitic fluid that was repeatedly negative for malignant cells. The ascites was considered most likely multifactorial, including portal hypertension from cirrhosis, hypoalbuminemia, and possible treatment-related liver injury. A third cycle of tislelizumab was administered with continued donafenib.

Imaging on April 29, 2024, showed slight hepatic progression, temporally associated with interruption of tislelizumab. Because each tislelizumab dose required hospital admission for intravenous administration and the patient declined further hospitalization, tislelizumab was discontinued after a total of 4 cycles; donafenib was continued without dose reduction. A third HAIC was performed on April 30, 2024. After the third HAIC and continuation of donafenib, subsequent imaging showed reduction of the hepatic target lesions.

At the most recent follow-up in July 2025 (19 months after diagnosis), imaging showed a residual hepatic lesion of approximately 8 mm without portal vein filling defect (Fig. 4A). Nasopharyngeal MRI showed mild mucosal thickening without overt mass; bilateral small cervical nodes were visible (Fig. 4B). HBV DNA was undetectable, and serial ALT/AST values had improved. The patient remained on entecavir and donafenib maintenance with an Eastern Cooperative Oncology Group performance status of 1.

**Figure 4.**
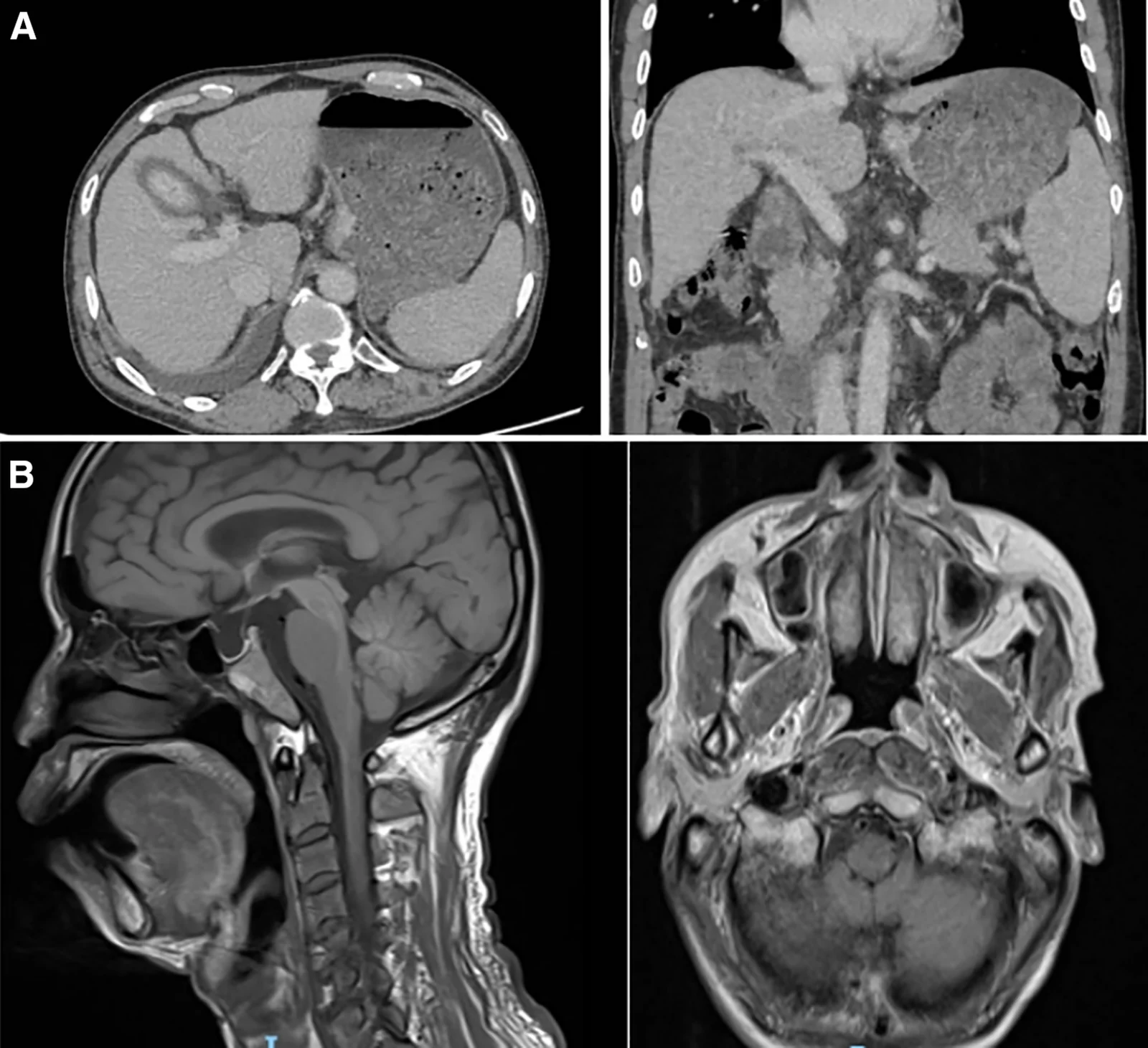
Latest follow-up imaging (July 2025). (A) Liver imaging showing a residual lesion of approximately 8 mm with no portal vein filling defect. (B) Nasopharyngeal imaging showing mild mucosal thickening of the posterior-superior wall without overt soft tissue mass, with bilateral small cervical lymph nodes; the NPC met radiographic complete response criteria during a tislelizumab-containing multimodality regimen without radiotherapy. NPC = nasopharyngeal carcinoma.

### 2.8. Patient perspective

In his own words, the patient reported: “After hepatic arterial infusion chemotherapy, I had poor appetite and nausea, but no other obvious discomfort.” He understood that radiotherapy could be used as salvage treatment if the nasopharyngeal tumor recurred and agreed to continue imaging surveillance and oral therapy.

### 2.9. Ethics approval and informed consent

This case report was approved by the Ethics Committee of the People’s Hospital of Guangxi Zhuang Autonomous Region (approval number: KY-ZC-2026-168; date: March 15, 2026). Written informed consent was obtained from the patient for publication of clinical details and images.

## 3. Discussion

### 3.1. Rarity and epidemiology

Synchronous HCC and NPC is extremely uncommon. Only 2 cases have been documented in the English-language literature.^[3,4]^ Multiple primary malignant neoplasm incidence among cancer survivors ranges from 2 to 17%, varying by tumor type, demographics, and follow-up duration.^[2]^ In East Asia, co-occurrence may be under-recognized because one malignancy can overshadow the other clinically.

### 3.2. Literature search strategy

PubMed and Web of Science were searched from inception through June 2025 using the terms “hepatocellular carcinoma,” “nasopharyngeal carcinoma,” “synchronous,” “multiple primary malignancy,” and “double primary cancer.” Reference lists of retrieved articles were screened. Reports were included if they described histologically confirmed synchronous or metachronous HCC and NPC. Two previously reported cases met these criteria.^[3,4]^

### 3.3. HBV, EBV, and etiological considerations

HBV is an established driver of HCC through viral DNA integration, oncogene activation, tumor suppressor inactivation, and chronic immune-mediated liver injury.^[6,11]^ Epidemiological studies also report associations between chronic HBV infection and extrahepatic cancers, but the evidence for a causal role of HBV in NPC is indirect and remains biologically plausible rather than confirmed.^[9,12]^ In this patient, the NPC was unequivocally EBV-associated, as shown by diffuse EBER expression. Serum VCA-IgA and EA-IgA were negative, but this finding does not establish that local EBV-driven oncogenesis was independent of systemic serological responses. Therefore, HBV should not be regarded as a proven common oncogenic driver for both tumors in this case.

### 3.4. Diagnostic approach

Diagnosis of synchronous double primary cancers requires histological distinction between tumors and exclusion of metastases (Warren and Gates criteria).^[13]^ Distinct IHC profiles (HCC: Glypican-3+/EBER−; NPC: P40+/EBER+) confirmed the diagnoses in this case. Patients with HCC in HBV-endemic regions should undergo head and neck evaluation including nasopharyngoscopy when indicated.

### 3.5. Therapeutic strategy and the role of immunotherapy

Managing synchronous HCC and NPC is challenging because treatment must control 2 histologically distinct tumors while limiting overlapping toxicity. First-line HCC treatment now includes atezolizumab plus bevacizumab or sintilimab plus a bevacizumab biosimilar (IMbrave150, ORIENT-32).^[14,15]^ For NPC, definitive radiotherapy-based treatment is standard for locoregionally advanced disease, with checkpoint inhibitors showing benefit in recurrent or metastatic disease.^[16,17]^

In this patient, a liver-directed approach was prioritized because portal vein tumor thrombus made HCC immediately life-threatening. HAIC is an accepted liver-directed treatment option for selected patients with unresectable HCC and portal vein invasion, particularly in Asia, but the comparative evidence is heterogeneous and depends on the HAIC regimen and comparator.^[18]^

The NPC achieved a radiographic complete response while the patient received a tislelizumab-containing multimodality regimen consisting of HAIC plus tislelizumab plus donafenib. Because these interventions were administered concurrently, the observed NPC response cannot be attributed solely to anti-programmed cell death protein 1 (PD-1) therapy.^[19–24]^

EBV-associated NPC may be responsive to PD-1 blockade, with objective response rates of 20 to 34% reported for anti-PD-1 monotherapy in recurrent or metastatic NPC.^[21,22]^ High PD-L1 expression and tumor-infiltrating lymphocytes in EBV-driven NPC provide a biological rationale for this observation.^[23]^

Donafenib may have immunomodulatory effects through VEGF inhibition and enhanced T-cell infiltration, but whether it synergized with tislelizumab in this individual patient cannot be determined.^[24]^

Post-treatment ascites with negative cytology was most likely multifactorial, including portal hypertension from cirrhosis, hypoalbuminemia, and possible treatment-related liver injury; negative cytology alone does not establish a drug-induced cause.

PD-L1 expression was not evaluated in the NPC specimen, and plasma EBV DNA was not measured. These biomarkers would be useful for assessing response and prognosis in similar cases.

### 3.6. Comparison with previously reported cases

The 2 previously reported cases illustrate the clinical heterogeneity of this rare dual malignancy.^[3,4]^ Yan et al reported a 60-year-old man who developed NPC 3 years after liver transplantation for HBV-related HCC, achieving sustained complete response after intensity-modulated radiotherapy (IMRT) and capecitabine plus nimotuzumab for lung metastases.^[3]^ Qi et al described a 56-year-old man with EBV-positive NPC in whom synchronous HCC was incidentally found on 68Ga-DOTATATE PET/MR.^[4]^ Chow et al reported that among 780 NPC survivors after definitive IMRT, liver cancer was among the second primary tumors with elevated risk (SIR 1.84; 95% CI: 1.37–2.42).^[25]^ None of these patients received combined HAIC, immunotherapy, and targeted therapy; our case is the first to describe this triple-modality approach in this setting (Table 1).

**Table 1:** Previously reported cases of synchronous/metachronous HCC and NPC.

Author/Year	Age/Sex	HBV	EBV	HCC (Stage/Tx)	NPC (Stage/Tx)	Syn/Met	Outcome
Yan et al 2012^[3]^	60/M	HBV-related HCC (OLT)	NR	HCC treated with OLT	T2N1M0 III; IMRT, then Cape + Nimotuzumab for lung mets	Metachronous (3 yr)	Sustained CR
Qi et al 2023^[4]^	56/M	NR	EBV + NPC	HCC (2 lesions), found on DOTATATE PET; NR	EBV + non-keratinizing NPC; chemotherapy	Synchronous	NR
Zhang (this report)	63/M	HBV DNA+	EBER + NPC; serum VCA-IgA/EA-IgA negative	BCLC C/CNLC IIIa; HAIC + anti-PD-1 + Donafenib	T3N2M0 III; anti-PD-1-containing multimodality therapy (RT deferred)	Synchronous	Alive, both controlled at 19 mo

BCLC = Barcelona Clinic Liver Cancer, CR = complete response, EBER = Epstein–Barr virus-encoded small RNA, HAIC = hepatic arterial infusion chemotherapy, HBV = hepatitis B virus, HCC = hepatocellular carcinoma, IMRT = intensity-modulated radiotherapy, NPC = nasopharyngeal carcinoma, NR = not reported, OLT = orthotopic liver transplantation, PD-1 = programmed cell death protein 1, RT = radiotherapy.

### 3.7. Limitations

This report is limited by its single-case design, the absence of PD-L1 testing and plasma EBV DNA monitoring, incomplete delivery of planned immunotherapy, the lack of radiotherapy for NPC, a relatively short 19-month follow-up, and the absence of comprehensive genomic profiling. Therefore, no causal or generalizable conclusions can be drawn.

## 4. Conclusion

Synchronous HCC and EBV-associated NPC is a rare clinical entity that warrants heightened vigilance in HBV carriers. In this patient, both tumors remained controlled during a tislelizumab-containing multimodality regimen, but the contribution of each treatment component cannot be determined from a single case. Definitive radiotherapy remains the standard of care for locoregionally advanced NPC. This case aligns with Sustainable Development Goal 3 by illustrating how multidisciplinary, integrated care may improve outcomes in patients with rare synchronous cancers; further studies are needed to define optimal management.

## Acknowledgments

The authors thank the patient for consenting to publication and the multidisciplinary clinical team involved in the patient’s care. No individual is named in this section.

## Author contributions

**Conceptualization:** Shijie Zhang.

**Data curation:** Shijie Zhang.

**Formal analysis:** Shijie Zhang.

**Funding acquisition:** Shijie Zhang.

**Investigation:** Shijie Zhang.

**Methodology:** Shijie Zhang.

**Project administration:** Shijie Zhang.

**Resources:** Shijie Zhang.

**Software:** Shijie Zhang.

**Supervision:** Shijie Zhang.

**Validation:** Shijie Zhang.

**Visualization:** Shijie Zhang.

**Writing – original draft:** Shijie Zhang.

**Writing – review & editing:** Shijie Zhang.
